# Analysis of mechanical properties and micro-mechanisms of concrete based on the method of using steel slag as aggregate replacement: Macro-mechanical tests and microscopic tests of four typical control groups

**DOI:** 10.1371/journal.pone.0352138

**Published:** 2026-07-23

**Authors:** Xiaobing Chen, Hengyi Yang, Jinhu Tong, Wei Hu, Fengyi Kang, Miao Zhang, Laiyuan Mao, Ronglong Zhao

**Affiliations:** 1 School of Transportation, Southeast University, Nanjing, Jiangsu, China; 2 Architects and Engineers Co., Ltd. of Southeast University, Nanjing, Jiangsu, China; 3 Nantong Highway Development Center, Nantong, Jiangsu, China; 4 Liaoning Provincial Transportation Development Center, Shenyang, Liaoning, China; 5 Wuxi Metro Operation Co., Ltd. Metro Building, Wuxi, Jiangsu, China; Jazan University College of Engineering, SAUDI ARABIA

## Abstract

There is still controversy over whether the mechanical properties of steel slag concrete are superior to those of ordinary crushed-stone concrete and which method of substituting steel slag for aggregates yields better mechanical performance. Four groups of specimens were designed in this study, namely ordinary crushed-stone concrete, coarse steel-slag natural-sand concrete, crushed-stone fine steel-slag concrete, and all-steel slag concrete. The compressive strength, flexural strength, splitting tensile strength and failure morphology of the four groups of specimens were tested. Meanwhile, microhardness, scanning electron microscopy, X-ray diffraction, and Fourier-transform infrared spectroscopy tests were conducted to analyze the differences in their mechanical properties from a microscopic perspective. The corresponding test results indicated that: (a) Under the test conditions of this study, the order of compressive strength, flexural strength, and splitting tensile strength of concrete is ordinary crushed-stone concrete < coarse steel-slag natural-sand concrete <crushed-stone fine steel-slag concrete < all-steel slag concrete. (b) Steel slag fine aggregates are superior to steel slag coarse aggregates in enhancing the mechanical properties of concrete. Crushing steel slag coarse aggregate into steel slag fine aggregate to replace ordinary fine aggregates is more effective in enhancing mechanical strength than directly using coarse steel slag to replace coarse aggregates. When steel slag simultaneously substitutes for both coarse and fine aggregates, the concrete strength is the highest. Despite limitations of this study, the above conclusions, if further validated by future studies, will provide valuable references for further research on steel slag concrete.

## 1. Introduction

Concrete is one of the most widely used construction materials worldwide, and it has become a crucial material in engineering construction applications [1]. However, due to over-exploitation, natural sand and gravel resources are becoming increasingly scarce [2]. Utilizing steel slag as a replacement for aggregates in concrete provides dual environmental benefits: it helps mitigate the depletion of natural sand and gravel reserves while simultaneously improving the overall utilization efficiency of steel slag [3–5]. At present, the recycling and resource utilization of industrial wastes has been advocated. In existing engineering applications, steel slag concrete has been widely used in pavement structures and non-load-bearing components of buildings [6].

Steel slag is a by‑product generated from iron ore processing during steelmaking. It exhibits higher strength than conventional crushed stone, together with a porous structure, angular shape, and relatively rough surface [7–8]. These physical properties not only enhance the aggregate strength of steel slag concrete but also improve the particle interlocking [9–10]. In addition, steel slag contains reactive components such as dicalcium silicate (C_2_S) and tricalcium silicate (C_3_S). When steel slag interacts with Portland cement and water, it undergoes secondary hydration reactions, which favor the formation of calcium silicate hydrate (C‑S‑H) gel. For instance, Ibrahim et al. confirmed via X‑ray diffraction analysis that steel slag promotes additional hydration and the formation of more C‑S‑H gel, leading to a denser microstructure [11]. Similarly, Chunlin et al. explicitly stated that steel slag can react with Ca(OH)_2_ in cement through secondary hydration, thereby altering the hydration products, microstructure, and pore characteristics, and consequently affecting the mechanical strength [12].

Currently, numerous studies have focused on the partial or full replacement of crushed stone aggregates in concrete with steel slag. For example, Saxena S. et al., Mitwally M. E. et al., and Saurabh K. et al. separately investigated the effects of steel slag as coarse aggregate on the mechanical properties and durability of concrete at various replacement ratios (0%, 10%, 30%, 50%, 75%, and 100%) [13–15]. These studies consistently demonstrated that when natural coarse aggregates are replaced by steel slag in steel slag concrete, the surface-active components in steel slag chemically react with water, thereby enhancing the strength and density of the interfacial transition zone (ITZ, the interface between the aggregate and the surrounding cement paste), thereby enhancing the strength and mechanical properties of the concrete. On the other hand, the use of steel slag as fine aggregate to replace natural fine aggregates for the production of steel slag fine aggregate concrete also represents an important research direction. For instance, Rehman S. et al., Darwis Z. et al., and Afaque M. et al. analyzed the influence of steel slag as fine aggregate on concrete strength and durability at replacement ratios ranging from 0% to 50% [16–18]. Their results indicated that the mechanical properties of concrete perform better when the replacement ratio of steel slag fine aggregate is within the range of 25%–50%. The substitution of natural fine aggregates with steel slag fine aggregates can also enhance concrete strength. This is mainly attributed to the fact that steel slag fine aggregate contributes to strengthening the cement mortar matrix in concrete and leads to a more compact microstructure.

In the studies mentioned above, some focus on the effects of steel slag as coarse aggregate on the mechanical properties of concrete, while others explore the role of steel slag as fine aggregate. However, studies that simultaneously use steel slag to replace both coarse and fine aggregates are relatively scarce. As a result, the existing literature mainly features three representative methods for preparing concrete by replacing natural aggregates with steel slag, namely concrete with 100% steel slag coarse aggregate replacement for crushed-stone, concrete with 100% steel slag fine aggregate replacement for natural sand, and concrete with complete steel slag replacement for both coarse and fine aggregates. Nevertheless, there is currently no consensus on which of the three replacement methods yields concrete with higher strength. In addition, it remains unclear whether the mechanical properties of steel slag concrete are superior to those of conventional crushed stone concrete under all conditions.

To address the above issues, four groups of comparative tests were designed under the same mix ratio conditions in this study, namely: ordinary crushed-stone concrete (B-B), coarse steel-slag natural-sand concrete with 100% volume replacement of crushed stone (B-SC), crushed-stone fine steel-slag concrete with 100% volume replacement of natural sand (B-SF), and all-steel slag concrete with both coarse and fine aggregates being fully replaced by steel slag (B-SCF). By conducting compression, bending, and splitting tensile strength tests on the four groups of specimens, their macroscopic mechanical properties were studied. Combined with failure morphology, microhardness, scanning electron microscopy (SEM), X-ray diffraction (XRD), and Fourier transform infrared spectroscopy (FTIR) tests, their microstructural characteristics were analyzed.

The mechanical property results of this comparative test show that the strength of the four groups of concrete from low to high is in the following order: B-B < B-SC < B-SF < B-SCF. This study has certain limitations. For example, under the same concrete preparation conditions, the water absorption rate of steel slag is significantly higher than that of crushed stone, leading to different effective water-cement ratios, which in turn causes differences in the compactness and workability of the four groups of specimens. In addition, although the test compared the strength of specimens at 180 days of age, the reactions of free calcium oxide (f-CaO) and free magnesium oxide (f-MgO) are relatively slow, and the long-term conclusions of this test still need further verification. Overall, this comparative test reveals the variation trend of short-term strength between steel slag concrete and conventional concrete to a certain extent. If the present findings are further validated by future studies, they will provide valuable references for future research on steel slag concrete.

## 2. Raw materials and test methods

### 2.1. Raw materials

#### 2.1.1. Cement.

The cement used in this study was ordinary Portland P•O 42.5 cement produced by the Jiangsu Jinfeng Company. Its technical specifications are listed in Table 1.

**Table 1:** Technical indices of the cement.

Material property	Units	Measured result
Density	g/cm^3^	3.06
Standard consistency water content	%	27.6
Initial setting time	min	216
Final setting time	min	272
Sulfur trioxide	%	1.98
Chloride ion content	%	0.011

The main mineral phases of cement were characterized by X-ray diffraction (XRD) using a Smartlab SE intelligent X-ray diffractometer. The scanning angle ranged from 5° to 70°, with a scanning rate of 10°/min. The obtained XRD data were processed and analyzed using Jade software, and the corresponding results are presented in Table 2.

**Table 2:** Mineral composition of cement.

Component	C_3_S	C_2_S	C_3_A	C_4_AF	CaSO_4_	MgO	Others
Content/%	65.52	8.65	11.96	9.24	3.63	3.29	9.79

#### 2.1.2. Steel-slag coarse aggregate.

The steel-slag coarse aggregate used in this study was steel converter slag with a size of 10–15 mm. It was provided by the Jiangsu Yonggang Group, and its technical specifications are provided in Table 3.

**Table 3:** Technical indices of the steel-slag coarse aggregate.

Material property	Units	Measured result
Apparent relative density	–	3.485
Crush value	%	13.4
Los Angeles wears loss	%	10.7
Flake particle content	%	10.1
Polished Stone Value (PSV)	–	52
10d water immersion expansion rate	%	0.87

#### 2.1.3. Steel-slag fine aggregate.

The particle size of the steel-slag fine aggregate used in this study was 0–5 mm. It was sourced from the same manufacturer as the coarse aggregate steel slag, and its technical specifications are listed in Table 4.

**Table 4:** Technical indices of the steel-slag fine aggregate.

Material property	Units	Measured result
Apparent relative density	–	3.408
Sand equivalent	%	95
Particles with a size of less than 0.075 mm.	s	2.0
Angular property	s	45

#### 2.1.4. Crushed stone.

The crushed stone used in this study was basalt from Hainan. It had a particle size of 10–15 mm, and its technical specifications are provided in Table 5.

**Table 5:** Technical indices of the basalt.

Material property	Units	Measured result
Apparent relative density	–	2.98
Crush value	%	10.4
Los Angeles abrasion loss	%	14.6
Flake particle content	%	9.8
Number of particles **<** 0.075 mm diameter	%	0.47
Adhesiveness	–	5
Polished stone value (PSV)	–	49

#### 2.1.5. Sand.

The sand used in this study was sourced from Suzhou, Jiangsu. It was classified as medium sand, and it had an apparent relative density of 3.509 and a fineness modulus of 3.0. Its specific technical indicators are listed in Table 6.

**Table 6:** Technical indices of the sand.

Material property	Units	Measured result
Apparent density	kg/m^3^	2,530
Bulk density	kg/m^3^	1,490
Porosity	%	41.1
Moisture content	%	0.02
Clay content	%	1.2
Mud block content	%	0.03

#### 2.1.6. Superplasticizer.

In this study, the S10 polycarboxylate superplasticizer (Suzhou Yisiter Building Materials Technology Co., Ltd.) was utilized. This material has a water-reduction rate of 22%, 1.5% of that of cementitious materials. Its technical specifications are listed in Table 7.

**Table 7:** Technical indices of the superplasticizer.

Material property	Units	Measured result
Density	kg/m^3^	1.016
PH value	–	6.4
Sodium sulfate content	%	0.2
Chloride ion content	%	0.05
Concrete water-reduction rate	%	22
Cement-paste fluidity	mm	208

### 2.2. Properties of concrete aggregates

#### 2.2.1. Appearance and natural gradation.

**Fig 1:**
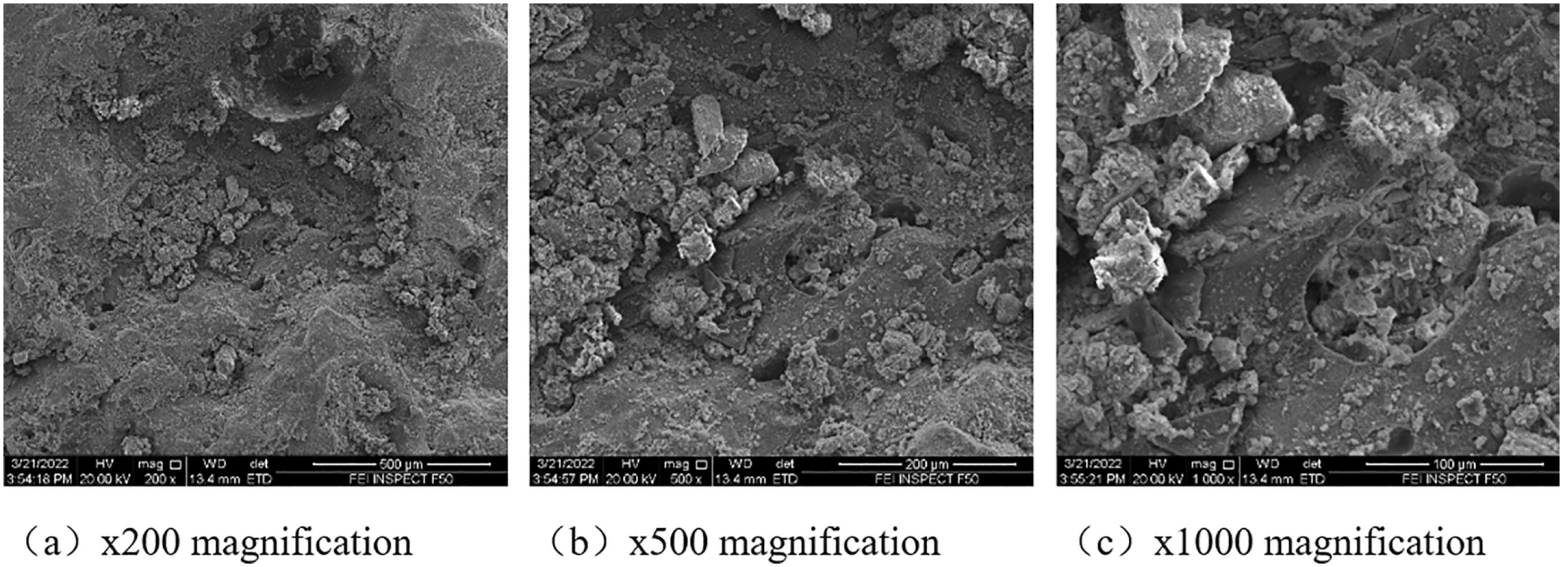
SEM surface morphology of steel slag aggregates.

Steel slag particles are blackish-brown in appearance, with good particle shapes. Their surfaces have a large number of pore structures and are relatively rough. This study used SEM to observe the microstructure of the surface of steel slag aggregates, as shown in Fig 1.

As observed from the SEM image in Fig 1, steel slag exhibits a rough and porous surface texture. The unique porous structure on the steel slag surface can provide sufficient space for cement paste penetration, enabling physical interlocking and thereby enhancing the interfacial adhesion and structural integrity between steel slag and cement paste [19].

**Fig 2:**
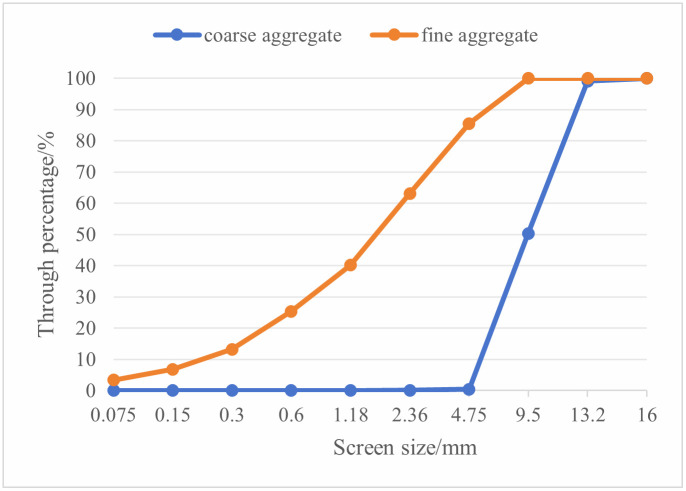
Steel slag aggregate gradation curves.

**Fig 3:**
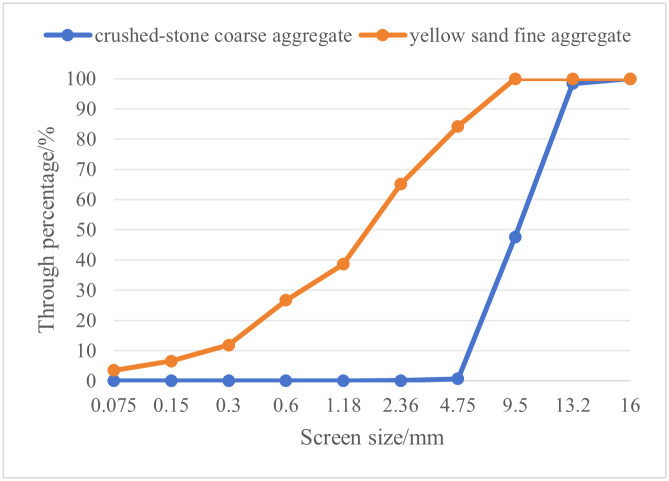
Natural aggregate gradation curves.

In accordance with the specification JTG 3432‑2024, the coarse and fine aggregates of steel slag were sieved respectively. The gradation curve of the steel slag aggregates is shown in Fig 2. The natural aggregates for this experiment are composed of basalt and natural sand, and the gradation curve of the natural aggregates is shown in Fig 3. It can be observed that the particle size distribution and gradation curves of natural aggregates and steel slag aggregates are similar. Therefore, the influence of aggregate gradation is expected to be limited. Both of the aggregates were in air-dry state.

#### 2.2.2. Chemical properties.

The chemical composition of steel slag aggregates was determined using a ThermoFisher ARL Perform’X 4200 X‑ray fluorescence (XRF) spectrometer, and the corresponding results are presented in Table 8.

**Table 8:** The chemical composition of steel slag aggregates.

Component	CaO	Fe_2_O_3_	SiO_2_	MgO	Al_2_O_3_	MnO	P_2_O_5_	Others
Content/%	40.52	20.05	15.16	4.84	3.63	3.29	2.72	9.79

#### 2.2.3. Volumetric stability analysis.

Free calcium oxide (f-CaO) in steel slag can react with water, leading to slight volume expansion of the steel slag. When incorporated into concrete, an excessive expansion rate may cause concrete expansion and cracking, which is detrimental to concrete durability and long-term stability. In accordance with standard YB/T 4328−2012, the f-CaO content of the steel slag was tested in this study. The results show that the f-CaO content is 2.82% for coarse steel slag and 1.71% for fine steel slag. In addition, the 10-day water immersion expansion rate of the steel slag was tested, and the test result was 0.87%, which meets the requirements of Standard GB/T 20491. These test results indicate that although the steel slag used in this study contains a certain amount of f-CaO, its content is within a stable range, and the steel slag aggregate is not prone to disintegration or severe uneven expansion.

### 2.3. Preparation of the steel-slag concrete specimens

#### 2.3.1. Basic concrete mixture design.

During this study, mixture design was conducted by using the equal-volume replacement method in specification JGJ 55–2011 [20]. The target strength of the selected concrete was C40. Multiple trials revealed that the benchmark water–cement ratio should be 0.36, the sand content should be 0.38, and the water-reducing agent should comprise 1.5% of the total amount of cementitious material. The basic mixture proportions are shown in Table 9.

**Table 9:** Basic mixture proportions (kg/m^3^).

Basalt	Sand	Cement	Water	Superplasticizer
1,135	696	428.5	154.3	6.43

#### 2.3.2. Design of the mixture proportions for the specimens.

Different steel slag substitution methods have different impacts on the mechanical properties of concrete. Further research is needed on specific approaches, such as replacing coarse aggregates, fine aggregates, or both. During this study, coarse steel-slag fully replaced crushed stone, and fine steel-slag fully replaced natural sand by equal volume. Four group of specimens were prepared according to the basic mixture proportions. These four groups of specimens are B-B, B-SC, B-SF and B-SCF respectively. The experimental material mixture proportions and slump data are provided in Table 10.

**Table 10:** Experimental mixture proportions for the specimens (kg/m^3^).

Group	Steel-slag coarse aggregate	Steel-slag fine aggregate	Basalt	Sand	Cement	Water	Super-plasticizer	Slump /mm
B-B	0	0	1,225.47	664.5	428.5	154.3	6.43	189
B-SC	1,433	0	0	664.5	428.5	154.3	6.43	175
B-SF	0	873.5	1,225.47	0	428.5	154.3	6.43	171
B-SCF	1,433	873.5	0	0	428.5	154.3	6.43	165

#### 2.3.3 Molding process.

**Fig 4:**
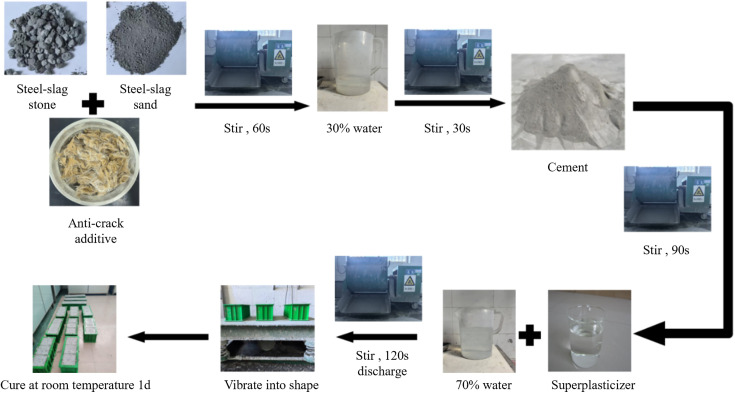
Cement concrete mixing and molding process. Authorized by Hengyi Yang and licensed under CC BY 4.0.

In this study, an SJD-60 cement concrete mixer was used to centrally mix the specimens with the same proportions at a speed of 42 r/min, as shown in the preparation process diagram in Fig 4. First, coarse and fine aggregates were added to the mixer and dry mixed for 60 s to ensure adequate mixing. Then, 30% of the water was added and mixed in for 30 s to moisten the aggregates. Next, cement was added and mixed in for 90 s so that it fully coated the aggregates. Finally, the superplasticizer and the remaining water were added, and the entire mixture was mixed for 120 s before it was discharged. The specimens were then compacted using vibration and cured at room temperature for one day. After demolding, they were moved to a standard curing room where they remained for a specified time. In the curing room, the curing temperature was held at 20 ± 2°C and a relative humidity greater than 95% was maintained.

### 2.4. Test methods

#### 2.4.1. Compressive-strength tests.

In this study, “Test Methods for Cement and Concrete for Highway Engineering” (JTG 3420−2020) [21] was referenced during the performance of the compressive-strength tests. For these tests, specimens were removed from the curing chamber after 7, 28, 90, and 180 days. These specimens had dimensions of 100 × 100 × 100 mm. The loading rate was set to 0.8 MPa/s, and it was maintained until the specimens failed. The failure load was recorded, and the average value from three specimens was used as the determined value. The compressive strengths of the specimens were then calculated according to Equation (1):


$$\begin{array}{c} f_{\text{cu}}=\eta \frac{F}{A} \end{array}$$ (1)

where *f*_cu_ represents the compressive strength of the specimen (MPa), *F* is the failure load (N), *A* denotes the compressed area (mm^2^), and η is the size conversion factor.

#### 2.4.2. Flexural-strength tests.

The flexural-strength tests were conducted according to JTG 3420−2020 [2 1]. After 28 days, these specimens were taken out of the curing room. The specimen dimensions were 100 × 100 × 400 mm. The loading rate was set to 0.08 MPa/s, and it was maintained until the specimens failed. The failure load was recorded, and the final value was the arithmetic mean of the failure loads of 3 specimens. The flexural strengths of the specimens were then calculated using Equation (2):


$$\begin{array}{c} f_{f}=\eta \frac{FL}{bh^{\text{2}}} \end{array}$$ (2)

where *f*_f_ represents the flexural strength of the specimen (MPa), *F* is the failure load (N), *L* denotes the span length (mm), *b* is the specimen width (mm), *h* represents the specimen height (mm), and η is the size conversion factor.

#### 2.4.3. Splitting tensile-strength tests.

The splitting tensile-strength tests were conducted according to standard T0560 as referenced in JTG 3420−2020 [21]. After 7, 28, 90, and 180 days, the specimens were taken out from the self-curing room and were tested using the WA-100C electro-hydraulic servo universal testing machine, which is produced by Wuxi New Luda Instrument Equipment Co., Ltd. For these tests, the specimen dimensions were 100 × 100 × 100 mm. The loading rate was set to 0.05 MPa/s, and the test continued until the specimens failed. The failure load was recorded, and the final value was the arithmetic mean of the failure loads of three specimens. The splitting tensile strength of the specimens were then calculated by Equation (3):


$$\begin{array}{c} f_{\text{ts}}=\eta \frac{\text{2}F}{\pi A}=\text{0}.\text{637}\eta \frac{F}{A} \end{array}$$ (3)

where *f*_ts_ represents the splitting tensile strength of the specimen (MPa), *F* is the failure load (N), *A* denotes the splitting surface area of the specimen (mm^2^), and η is the size conversion factor.

#### 2.4.4. Microhardness tests.

**Fig 5:**
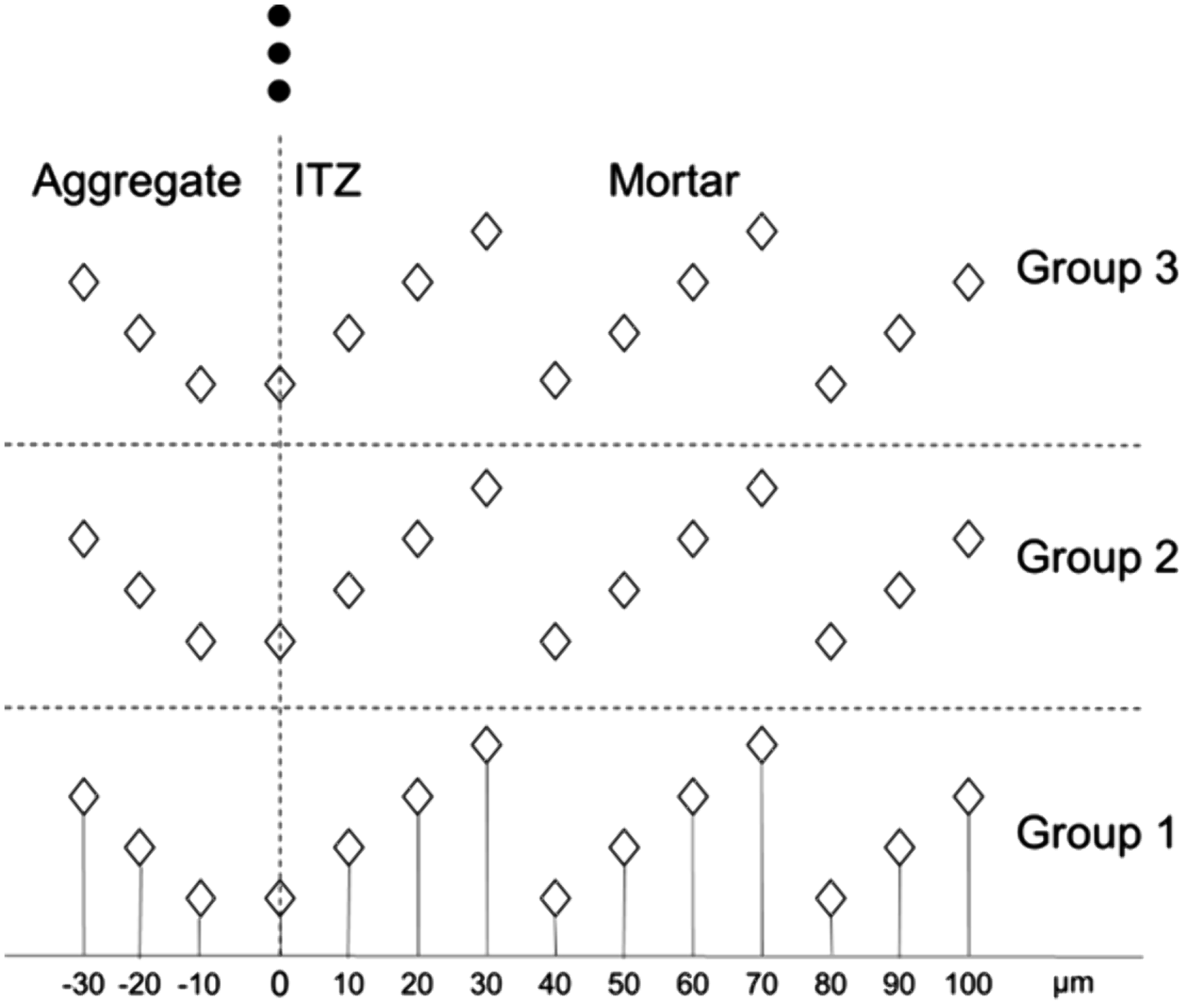
Microhardness test grid.


After specimens were cut and polished into 1 cm^3^ cubic samples, the FM-ARS900 microhardness tester was used to obtain their microhardness values. Due to the significant difference in strength between aggregates and cement paste, different test loads were adopted in the experiment: a load of 300 gf was used for testing the cement paste area, and a load of 1000 gf was used for testing the aggregate area. The holding time was 10 s. The microhardness test grid is shown in Fig 5. First, the interface between the aggregate and the paste was identified and the first point was placed at this location. Then, the next point was placed at a distance of 10 μm from the first point in the aggregate direction. Since the aggregate section was uniform, if no dispersion occurred during testing, three points were placed in the aggregate region. Then, another point was placed near the interface but on the paste side; this point was shifted by 10 μm horizontally and by some larger amount vertically, thereby ensuring that the second point did not affect the first point. Due to the considerable variation in the strength of the cement paste, the determined value was the arithmetic mean of five measurement values. The microhardness was then calculated according to Equation (4):

$$\begin{array}{c} HV=0.102\times \frac{F}{S}\times 2F\frac{\left(\frac{\theta }{\text{2}}\right)}{d^{\text{2}}}=0.1891\frac{F}{d^{\text{2}}} \end{array}$$ (4)

where *S* represents the indentation surface area (mm^2^) and *S* = d^2^/(2sin(θ/2)), *F* denotes the test load (N), *d* is the arithmetic mean of the indentation diagonals (mm), *θ* represents the diamond-indenter face angle (°), which in this study was 136°, and *a* is a coefficient, which was taken as 0.102 according to national standard GBT 4340.1−2009.

#### 2.4.5. Scanning Electron Microscopy tests.

For these analyses, specimens were prepared as 1 cm^3^ cubes through cutting and polishing. Subsequently, a Q150TS high-resolution sputter coater was employed to apply a gold coating. The samples were then examined using an FEI Inspect F50 scanning electron microscope. Each sample underwent imaging at three distinct surface locations, utilizing magnifications of ×1,000, × 2,000, and ×5,000. From these images, one representative area per sample was chosen for further evaluation.

#### 2.4.6. X-Ray Diffraction tests.

In this study, sample fragments were ground into a powder using a sealed-type sample-preparation grinder, after which they were sieved through a 0.075 mm mesh. Finally, they were dried in an oven at 105°C for 4 h. An intelligent X-ray diffractometer, the Smartlab SE, manufactured by Rigaku (Japan), was used for the analysis. It employed a step scan mode with an angle range of 5–70° and a scanning speed of 2°/min. The XRD data were processed and analyzed using the Jade software.

#### 2.4.7. Fourier-Transform Infrared Spectroscopy tests.

Sample powders were thoroughly mixed and ground with dry potassium bromide (KBr), and the resultant powder mixtures were pressed into semi-transparent circular pellets using a mold. Analysis was performed using a Nicolet IS10 Fourier-transform infrared spectrometer manufactured by Thermo. The testing wave-number range was 400–4,000 cm^-1^ and the resolution was 2 cm^-1^. The data were processed and analyzed using the OMINIC software.

## 3. Analysis of the mechanical properties

In the compressive strength test, flexural strength test, and splitting tensile strength test, in accordance with the JTG 3420−2020 [21], each group was tested in triplicate, and the strength values of steel slag concrete were presented as the average value (AV) and standard deviation (SD). In addition, the coefficient of variation was calculated to evaluate the relative dispersion of the experimental data.

### 3.1. Compressive-strength analysis

**Fig 6:**
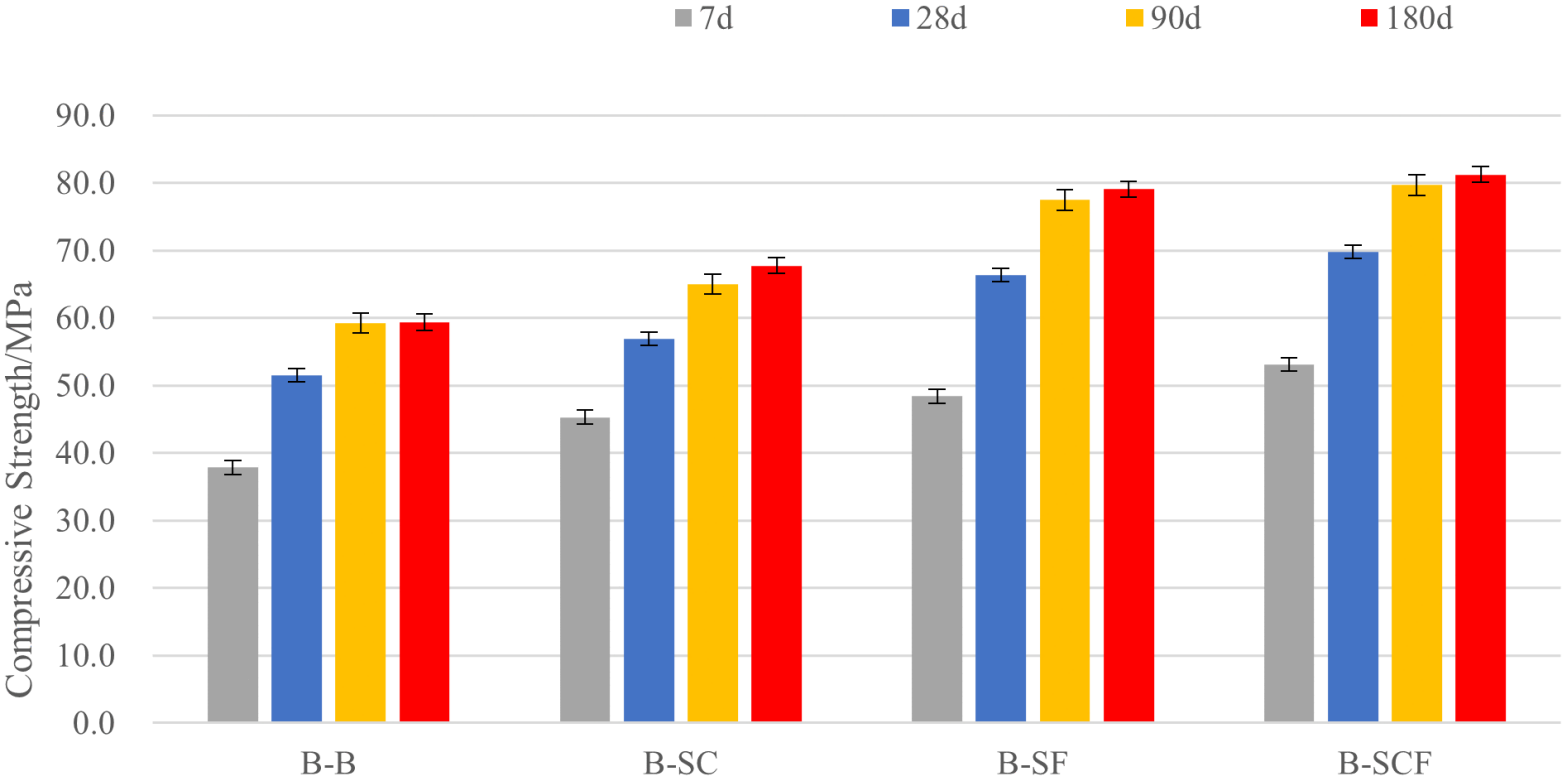
Variations in the compressive strength of specimens.

The compressive-strength test results for concrete cured for 7, 28, 90, and 180 days are presented for the four groups of specimens. By setting the four groups in Table 11 as independent variables and the compressive strength as the dependent variable, the variations in the compressive strengths of concrete of different ages were plotted, as shown in Fig 6. Raw data was shown in S2 Table. Statistical analysis revealed that the calculated coefficients of variation were less than 10%, indicating low data dispersion and acceptable reliability of the test results.

**Table 11:** Compressive strengths of specimens.

Group	Day 7 compressive strength/MPa		Day 28 compressive strength/MPa		Day 90 compressive strength/MPa		Day 180 compressive strength/MPa	
	AV	SD	AV	SD	AV	SD	AV	SD
B-B	37.9	0.07	51.5	0.44	59.3	1.19	59.4	0.25
B-SC	45.3	0.70	56.9	0.56	65.0	0.57	67.7	1.25
B-SF	48.4	0.46	66.3	0.53	77.5	0.81	79.1	0.55
B-SCF	53.1	0.63	69.8	0.65	79.7	0.26	81.2	0.78

The results in Table 11 and Fig 6 indicate that the compressive strength of the B-SC, B-SF, and B-SCF specimens of various ages were all greater than that of the B-B. The day 28 compressive strength of the B-B was 51.5 MPa, while the day 28 compressive strengths of the B-SC, B-SF, and B-SCF were 56.9 MPa, 66.3 MPa, and 69.8 MPa, respectively; these values represent increases of 10.2%, 28.8%, and 35.3%, respectively. These results indicate that, for the same water–cement ratio, replacing various types of aggregates with steel slag can enhance the compressive strengths of concrete of various ages. In addition, the compressive-strength improvements that were produced by utilizing steel-slag fine aggregate were significantly greater than those that were produced by utilizing steel-slag coarse aggregate.

### 3.2. Flexural-strength analysis

**Fig 7:**
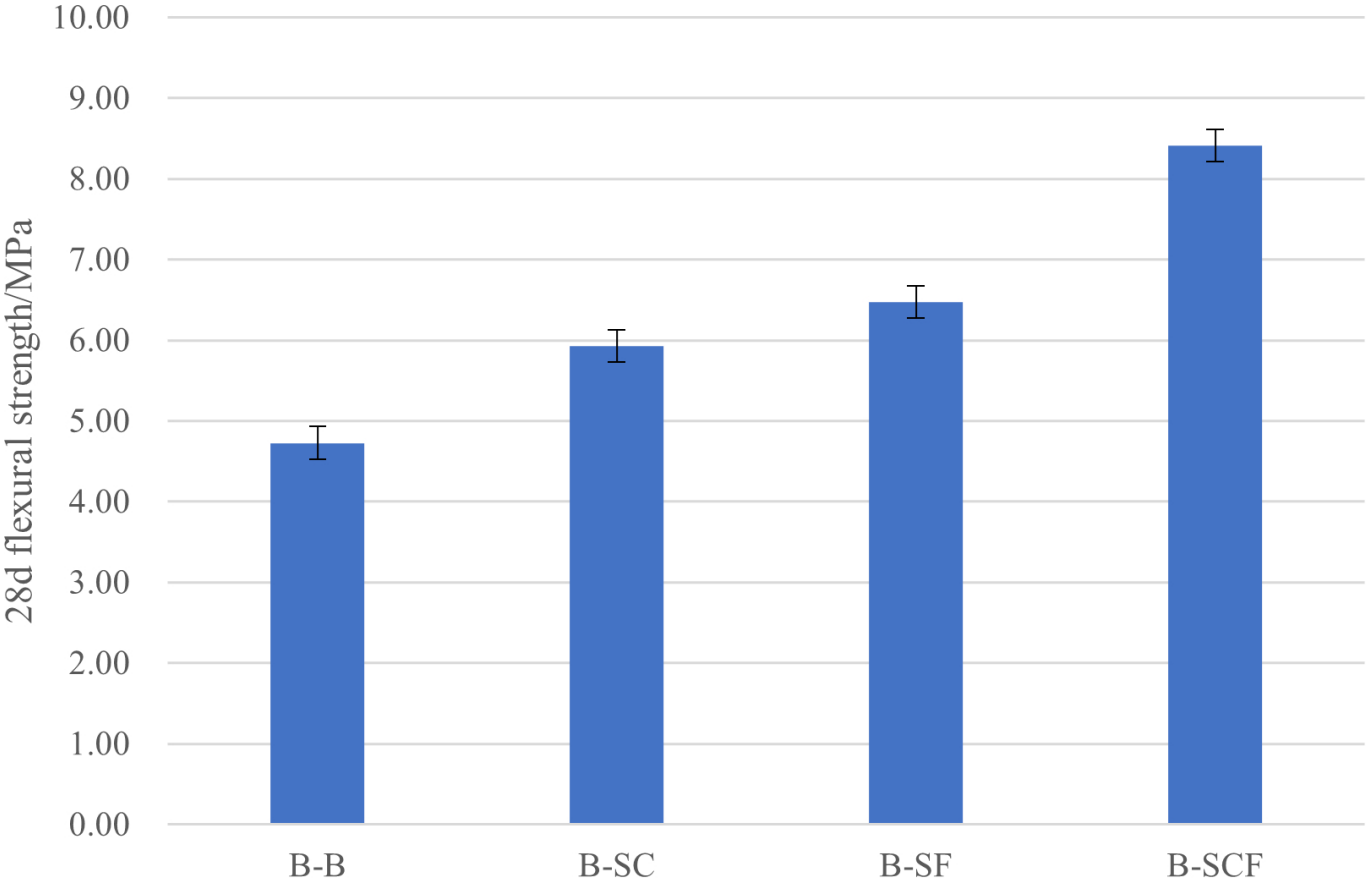
Variations in the flexural strength of specimens.

The day 28 flexural-strength test results for the four groups are presented in Table 12. By setting the four groups from Table 12 as independent variables and the day 28 flexural strength as the dependent variable, the variation pattern of the day 28 flexural strength was plotted, as shown in Fig 7. Raw data was shown in S2 Table. Statistical analysis revealed that the calculated coefficients of variation were less than 10%, indicating low data dispersion and acceptable reliability of the test results.

**Table 12:** Day 28 flexural strengths of specimens.

Group	Day 28 flexural strength/MPa	
	AV	SD
B-B	4.73	0.14
B-SC	5.93	0.27
B-SF	6.47	0.20
B-SCF	8.41	0.05

Fig 7 shows that the day 28 flexural strengths of the B-SC, B-SF, and B-SCF were greater than that of the B-B. The day 28 flexural strength of the B-B was 4.73 MPa, while those of the B-SC, B-SF, and B-SCF were 5.93 MPa, 6.47 MPa, and 8.41 MPa, respectively; these values represent increases of 25.4%, 36.8%, and 77.8%, respectively. These results indicate that replacing various aggregate types with steel slag can enhance the flexural strength of concrete, and that steel-slag fine aggregate provides a better improvement effect on the flexural strength than steel-slag coarse aggregate.

### 3.3 Splitting tensile-strength analysis

**Fig 8:**
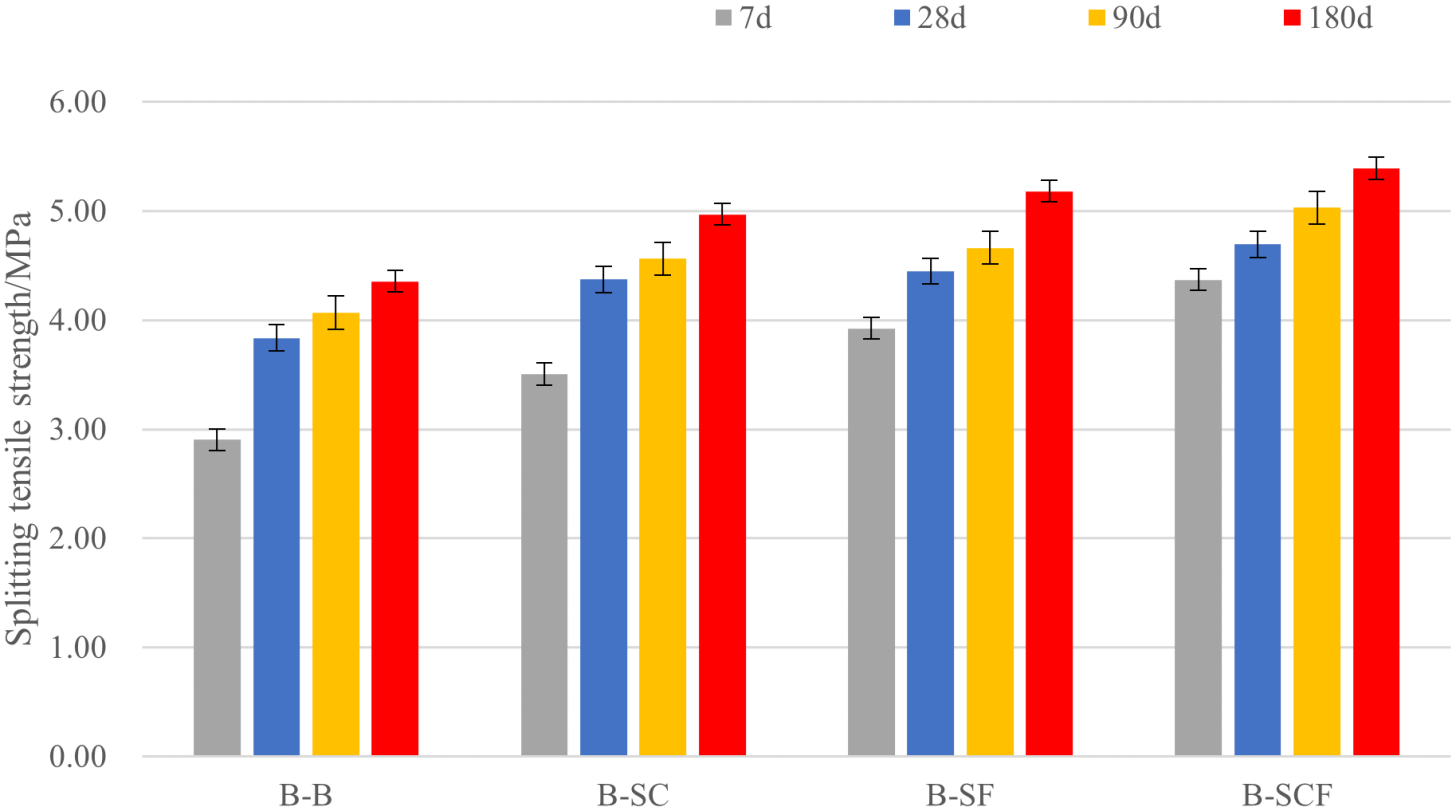
Variations in the splitting tensile strength of specimens.

Table 13 shows the splitting tensile strength values of concrete specimens after being cured for 7, 28, 90, and 180 days. By setting the four groups in Table 13 as independent variables and the splitting tensile strength as the dependent variable, the variation patterns of the splitting tensile strength of concrete of various ages were plotted, as shown in Fig 8. Raw data was shown in S2 Table. Statistical analysis revealed that the calculated coefficients of variation were less than 10%, indicating low data dispersion and acceptable reliability of the test results.

**Table 13:** Splitting tensile strength of concrete specimens.

Group	Day 7 splitting tensile strength/MPa		Day 28 splitting tensile strength/MPa		Day 90 splitting tensile strength/MPa		Day 180 splitting tensile strength/MPa	
	AV	SD	AV	SD	AV	SD	AV	SD
B-B	2.90	0.15	3.83	0.34	4.07	0.12	4.35	0.13
B-SC	3.50	0.13	4.37	0.15	4.56	0.07	4.97	0.14
B-SF	3.92	0.09	4.45	0.15	4.66	0.10	5.18	0.19
B-SCF	4.37	0.16	4.69	0.08	5.03	0.12	5.39	0.20

The results in Table 13 and Fig 8 indicate that the splitting tensile strength of the B-SC, B-SF, and B-SCF specimens of various ages were all greater than that of the B-B. The day 28 splitting tensile strength of the B-B was 3.83 MPa, compared with 4.37 MPa, 4.45 MPa, and 4.69 MPa for B-SC, B-SF, and B-SCF, respectively; these values represent increases of 14.10%, 16.19%, and 22.45%, respectively. The findings suggest that the replacement of various types of aggregates with steel slag can enhance the splitting tensile strength of concrete across various curing ages. Furthermore, steel-slag fine aggregate produces slightly better enhancements to the splitting tensile strength of concrete than steel-slag coarse aggregate.

### 3.4. Failure morphology analysis

**Fig 9:**
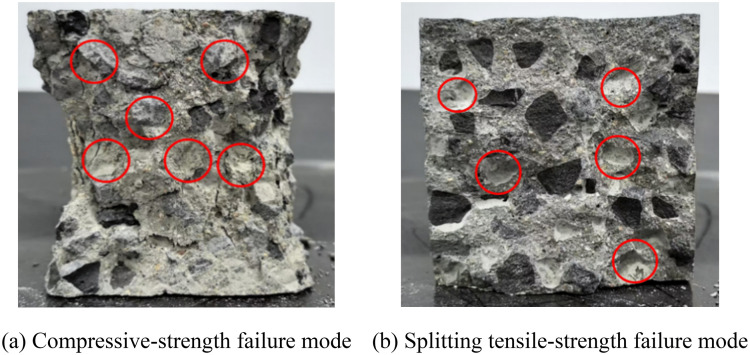
Failure modes observed after strength testing of B-B specimens. (a) Compressive-strength failure mode (b) Splitting tensile-strength failure mode. Authorized by Hengyi Yang and licensed under CC BY 4.0.

**Fig 10:**
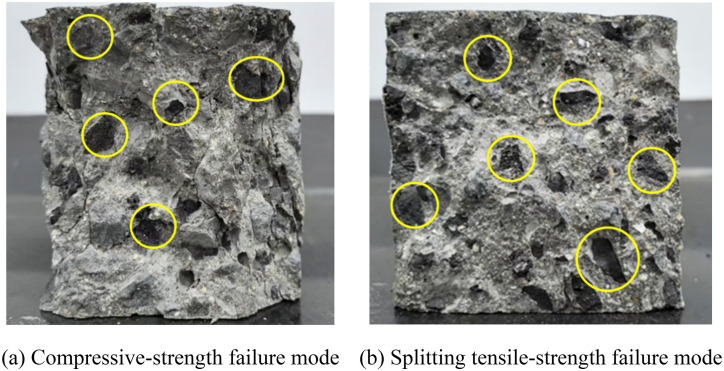
Failure modes observed after strength testing of B-SC specimens. (a) Compressive-strength failure mode (b) Splitting tensile-strength failure mode. Authorized by Hengyi Yang and licensed under CC BY 4.0.

**Fig 11:**
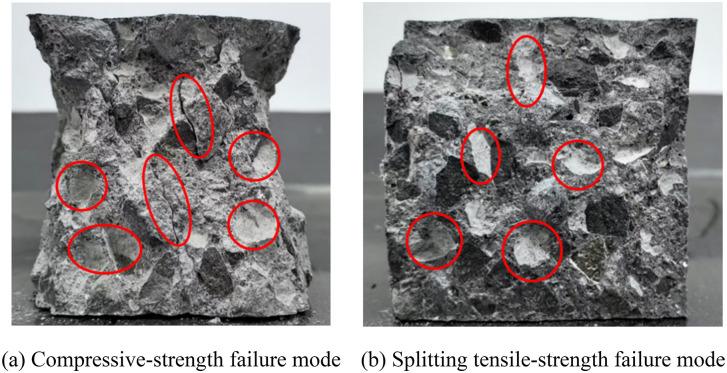
Failure modes observed after strength testing of B-SF specimens. (a) Compressive-strength failure mode (b) Splitting tensile-strength failure mode. Authorized by Hengyi Yang and licensed under CC BY 4.0.

**Fig 12:**
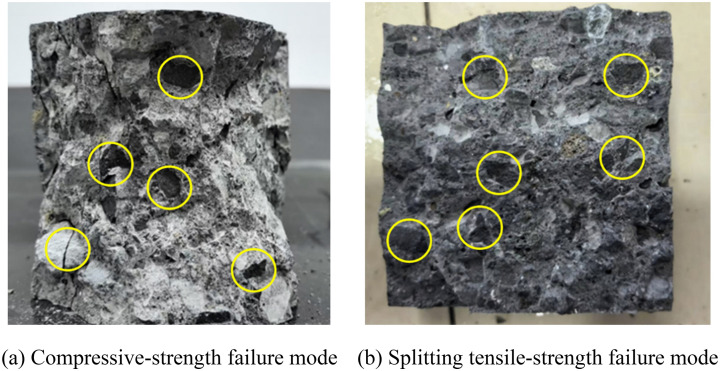
Failure modes observed after strength testing of B-SCF specimens. (a) Compressive-strength failure mode (b) Splitting tensile-strength failure mode. Authorized by Hengyi Yang and licensed under CC BY 4.0.

Representative compression and splitting tensile strength failure specimens from the four groups were selected for analysis, which was performed to study the effects of different steel-slag replacement methods on the failure morphologies of concrete specimens. The failure morphologies of the four groups of specimens are shown in Figs 9–12.

As can be seen in Fig 9, the compressive-strength failure mode was a typical cone failure. Cracks tended to initiate near the interface where the aggregate bonds with the cement paste, and in this test, many exposed intact aggregates and the separation surface between the cement paste and aggregate were observable. The splitting tensile-strength specimens were split into two after failure; in addition to failure of the crushed-stone aggregates at the fracture surface, significant damage to the cement paste–aggregate bonding interfaces also occurred. The X-CT test result is shown in Fig A in S1 Text.

Fig 10 shows that the compressive-strength and splitting tensile-strength specimens of the B-SC had a lighter cement-paste color than the B-B specimens. The compressive-strength failure mode was characterized by a top cone failure. Minimal damage occurred at the bonding interface between the steel-slag aggregates and the cement paste, but some damage to the steel slag aggregate can be observed, as well as noticeable cracks in the cement paste body. After the splitting tensile-strength specimen failed, it split into two parts. In addition to the visible separation failure that occurred between the cement paste and the steel-slag aggregates at the fracture surface, damage to the steel-slag aggregates was also observed.

Fig 11 shows that the failure mode of the compressive-strength specimen was characterized by typical conical failure, and most of the damage occurred at the cement paste–aggregate bonding interface. A significant number of exposed intact crushed-stone aggregates and separation surfaces between the cement paste and aggregates were observed, along with damage cracks at the cement paste–aggregate bonding interface. The splitting tensile-strength specimens broke into two pieces upon failure; on the fracture surface, in addition to visible damage to the crushed-stone aggregates, substantial damage was also observed at the bonding surfaces between the cement paste and the crushed-stone aggregates.

Fig 12 shows that the color of the cement paste in the compressive-strength and splitting tensile-strength specimens of B-SCF was relatively dark. The compressive-strength failure mode was characterized by a cone failure at the top, while minimal damage occurred at the bonding interface between the steel-slag aggregate and the cement paste. A significant amount of damage to the steel-slag aggregate was observed; in fact, visible cracks appeared in the steel-slag aggregate. In the splitting tensile-strength specimens, the damage resulted in a clean split. In addition to damage at the cement paste–steel-slag aggregate bonding interface, considerable damage to the steel-slag aggregate was also observed. The X-CT test result is shown in Fig B in S1 Text.

The results in Figs 912 indicate that cracks in ordinary concrete usually occur at the interface. When crushed stone was replaced with steel-slag coarse aggregate, minimal damage occurred at the cement paste–steel slag bonding interfaces in the specimens; however, a significant amount of damage to the steel-slag aggregate itself was observed. The possible reason is that replacing crushed stone with steel-slag coarse aggregate can enhance the strength of the transition zones at the interfaces between the aggregates and the cement paste. Cracks can only occur at the weak points between steel slag aggregates and cement paste. Due to the differences in mechanical properties of individual coarse steel slag aggregates, the cracks in this test occurred on the coarse steel slag pieces that were prone to breakage. In addition, when natural sand was replaced with steel-slag fine aggregate, the color of the cement paste changed from light to dark. This was likely caused by the reaction between the steel slag fine aggregate and the cement, which produced hydration products. The failures in the specimens predominantly occurred at the cement paste–crushed stone bonding interfaces, which might indicate that replacing natural sand with steel slag fine aggregate can improve the overall strength of the cement paste. Cracks can only occur at the weak points of natural crushed stones and interfaces. Due to the high strength of the concrete crushed stones in this test, most of the cracks appeared at the interfaces. All-steel slag concrete comprehensively enhances the strength of coarse aggregates, cement paste and interfaces. As the mechanical properties of individual coarse steel slag aggregates are different, cracks mostly occur on the coarse steel slag pieces that were prone to breakage.

## 4. Microstructural performance testing

To investigate the microscopic mechanisms underlying the differences in mechanical properties resulting from the partial or complete substitution of traditional concrete aggregates with steel slag. Microhardness, SEM, XRD, and FTIR tests were conducted with day 28 specimens from the four groups during this study. These analyses examined the means by which utilizing steel slag rather than traditional aggregates affected the microhardness, microstructure, element distribution, hydration products, and functional group composition in the resultant concrete.

### 4.1. Microhardness analysis

**Fig 13:**
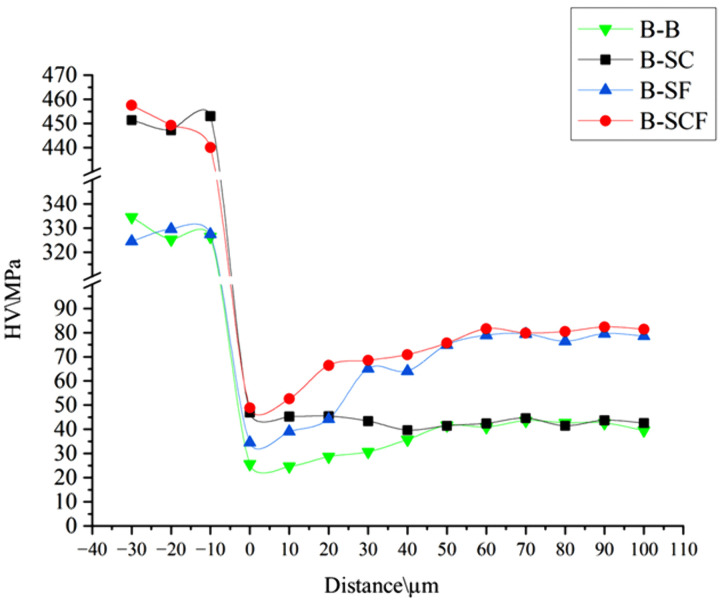
Variations in the microhardness values of specimens.

In the coarse aggregate part, it can be seen from Fig 13 that the microhardness values of B-B and B-SF are relatively close, with the microhardness of crushed stone ranging from 320 to 340 MPa. Meanwhile, the microhardness values of B-SC and B-SCF are relatively close, with the microhardness of steel slag ranging from 440 to 460 MPa, indicating that the microhardness of steel slag is higher than that of crushed stone. In the cement paste part, the microhardness of the four groups of specimens mostly shows a slight upward trend with the increase of distance. Among them, the microhardness values of B-B and B-SC, B-SF and B-SCF are respectively similar. After the microhardness of the cement paste tends to be stable, the microhardness of B-B specimens is 35 ~ 43 MPa, that of B-SC is 39 ~ 45 MPa, that of B-SF is 65 ~ 80 MPa, and that of B-SCF is 66 ~ 82 MPa, with the order from high to low being B-SCF > B-SF > B-SC > B-B. In the ITZ, the valley values of microhardness in descending order are B-SCF (48.8 MPa), B-SC (46.9 MPa), B-SF (34.5 MPa), and B-B (25.6 MPa). The reason may be that the surface-active phases in both steel slag coarse aggregate and steel slag fine aggregate can undergo hydration reactions, making the surrounding areas denser and thus improving the microhardness of the adjacent areas. Due to the larger specific surface area of steel slag aggregate, it has a more significant effect on enhancing the hardness of its surrounding areas. Therefore, the microhardness of the ITZ shows B-SC > B-SF, while in the cement paste zone, the microhardness shows B-SF > B-SC. The microhardness results are consistent with the macroscopic mechanical behaviour.

### 4.2. Scanning Electron Microscopy analysis

**Fig 14:**
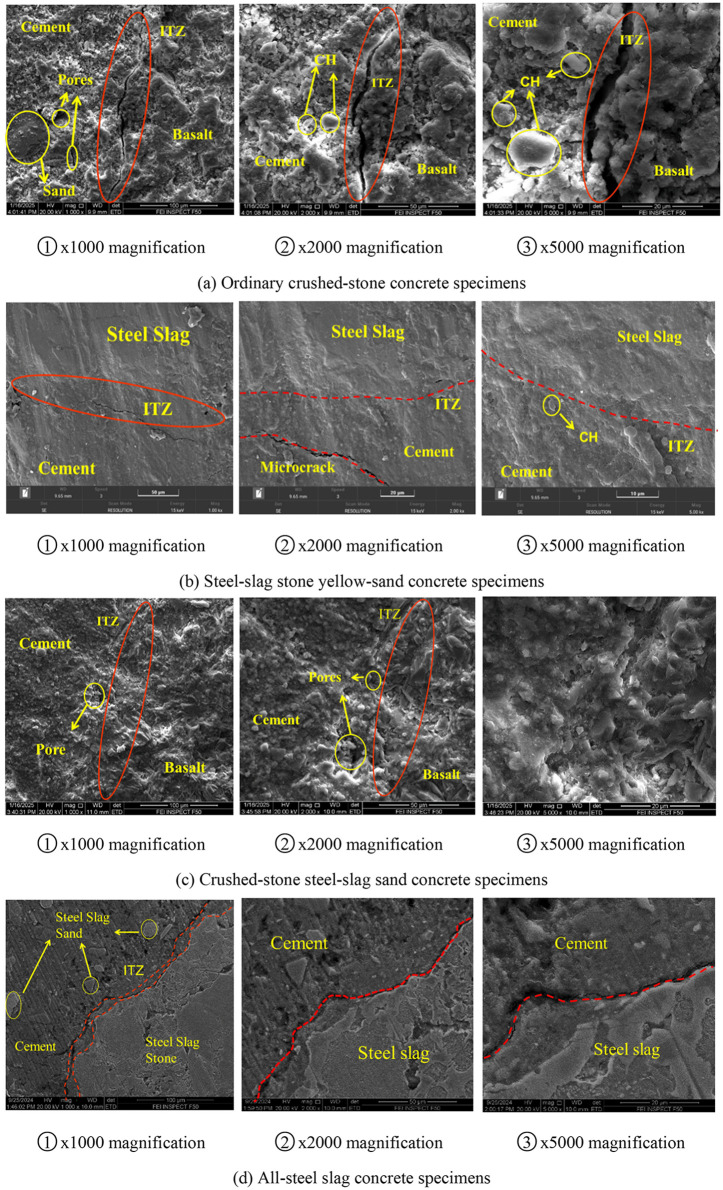
Microstructural morphologies of specimens. (a) B-B specimens, with the magnification of x1000, x2000, x5000. (b) B-SC specimens, with the magnification of x1000, x2000, x5000. (c) B-SF specimens, with the magnification of x1000, x2000, x5000. (d) B-SCF, with the magnification of x1000, x2000, x5000.

Fig 14 shows that the B-B specimens had more pores in the cement paste than the other specimens, indicating lower densities and hardness values. The ITZ displayed significant separating cracks, along with plate-like Ca(OH)_2_ and needle-like ettringite fillings. These findings may suggest that the hydration at the cement paste–aggregate interface was insufficient, which led to limited hydration products available for filling; thus, there was a lower bond strength at the interface. In the B-SC specimens, the steel slag was tightly bonded with the cement paste, and no separating cracks were apparent. As the magnification increased, no obvious separation surfaces appeared; however, microcracks were present in the cement paste. The possible reason is that the hydration products generated by the hydration reactions between the surface-active substances in the steel slag and the cement paste evenly filled the pores in the ITZ, thereby enhancing the strength of this zone. These hydration products also adhere to the rough surfaces of the steel slag, thereby providing effective mechanical interlocking and strengthen the bonds between the steel slag and the cement paste. This observation may explain why the B-SC specimens produced greater improvements in the splitting tensile and flexural strengths than on the compressive strength, as was observed in the macroscopic tests. The tightness of the ITZ in the B-SF specimens was between that of the B-B and B-SC specimens. Pores were present in the transition zone of the B-SF specimens, but the cement-paste portion was tighter and smoother, with no pores or cracks, and it was better filled with hydration products. Might be similar to steel-slag coarse aggregate, steel-slag fine aggregate dispersed in the cement paste reacted with surrounding cementitious materials, and the resulting hydration products filled voids, increasing paste strength. In the B-SCF specimen, both coarse and fine aggregates were replaced by steel-slag, yielding a dense, smooth ITZ and cement paste with no visible pores or cracks.

**Fig 15:**
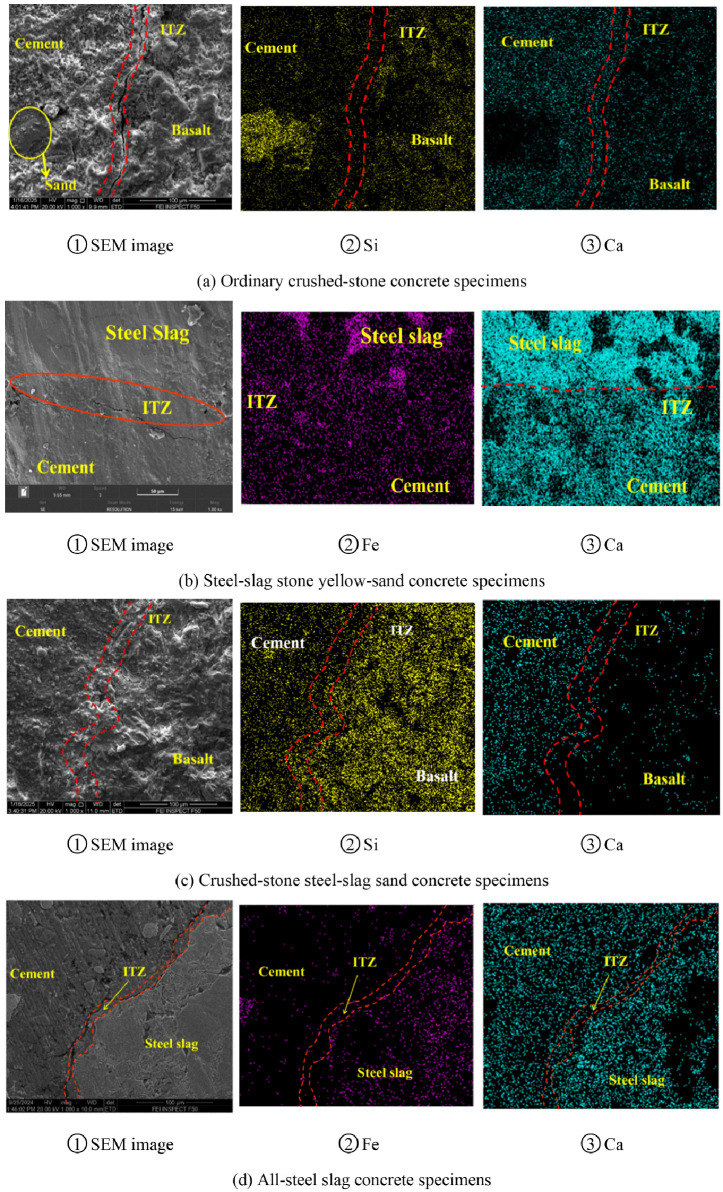
Elemental distribution of specimens with x1000 magnification. (a) B-B specimens, with SEM image and elemental distribution of Si and Ca. (b) B-SC specimens, with SEM image and elemental distribution of Fe and Ca. (c) B-SF specimens, with SEM image and elemental distribution of Si and Ca. (d) B-SCF specimens, with SEM image and elemental distribution of Fe and Ca.

The elemental distribution of the four groups of specimens is shown in Fig 15. Among them, Fe and Ca were analyzed for the B-SC and B-SCF control groups, while Si and Ca were analyzed for the B-B and B-SF groups since there was no steel slag coarse aggregate.

As shown in Fig 15, based on the distribution characteristics of iron elements, the ITZ between the steel slag and the cement paste can be clearly distinguished in the B-SC and B-SCF specimens. The steel slag phase had a significantly higher Fe content, while the cement paste phase had a relatively lower Fe content. According to the distribution characteristics of Si and Ca, the ITZ between the crushed-stone and the cement paste can be clearly distinguished in the B-B and B-SF specimens. The crushed-stone phase had a significantly higher Si content and a lower Ca content, while the cement paste phase had a lower Si content but a higher Ca content. In the Ca distribution map, compared with the B-B and B-SF specimens, the B-SC and B-SCF specimens had a denser Ca distribution in the ITZ. However, the B-B and B-SF specimens had a relatively sparse distribution. The possible reason is that the active substances (f-CaO) on the surface of the steel slag react with water to produce Ca(OH)_2_, and part of the Ca(OH)_2_ further undergoes hydration to form C-S-H gel, which fills the ITZ, resulting in a higher calcium content in the B-SC and B-SCF specimens at this location.

### 4.3. X-Ray Diffraction analysis

**Fig 16:**
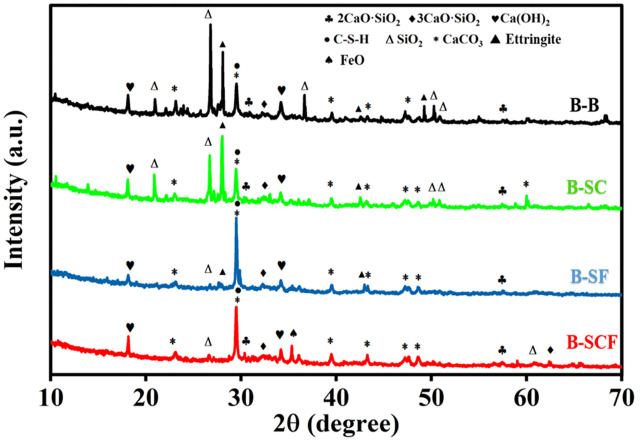
X-Ray Diffraction patterns for specimens.

After conducting XRD analyses with four groups of specimens, the resulting XRD spectra were plotted, as shown in Fig 16.

Fig 16 shows that, after hydration, the chemical compositions of the four groups of specimens primarily included calcium hydroxide (Ca(OH)_2_), calcium carbonate (CaCO_3_), calcium silicate hydrate (C-S-H), and ettringite. The unreacted quartz (SiO_2_), iron oxide (FeO), dicalcium silicate (2CaO·SiO_2_), and tricalcium silicate (3CaO·SiO_2_) were also included.

The B-SC, B-SF, and B-SCF specimens exhibited weaker Ca(OH)_2_ diffraction peaks at 2θ = 34.1° than the B-B specimen; however, they exhibited stronger characteristic peaks than the B-B specimen at the overlapping regions of CaCO_3_ and C-S-H. This might indicate that the replacement of traditional aggregates by steel slag was associated with the formation of C-S-H, and that the steel-slag fine aggregate was more effective than the steel-slag coarse aggregate. It also might suggest that the substitution of crushed-stone and natural sand with steel slag reduced the Ca(OH)_2_ and CaCO_3_ proportions in the concrete. The B-B and B-SC specimens showed strong quartz diffraction peaks at 2θ = 26.7°, but the diffraction peak of the B-SC specimen had a smaller intensity than that of the B-B specimen. This result might indicate that the addition of steel slag was associated with the consumption of SiO_2_. Ettringite diffraction peaks appeared in B-B, B-SC, and B-SF spectra, and the intensity of the B-SF peak was significantly lower than that of the B-B and B-SC peaks. The possible reason is that the addition of steel-slag fine aggregate effectively suppressed the formation of ettringite. The formation of ettringite requires a certain amount of space, and the addition of steel-slag fine aggregate caused the microstructure of the cement paste to be more compact, which reduced the pore size and therefore the quantity of ettringite. The chemical reactions that produce ettringite in concrete are shown in Equations (5) and (6):


$$\begin{array}{c} {\text{C}}_{\text{3}}\text{A }+\;\text{3}(\text{CaS}{\text{O}}_{\text{4}}\cdot \text{2}{\text{H}}_{\text{2}}\text{O})\;+\text{ 2Ca}(\text{OH}{)}_{\text{2}}\;+\text{ 24}{\text{H}}_{\text{2}}\text{O }=\text{ 3CaO}\cdot \text{A}{\text{l}}_{\text{2}}{\text{O}}_{\text{3}}\cdot \text{3CaS}{\text{H}}_{\text{4}}\cdot \text{32}{\text{H}}_{\text{2}}\text{O} \end{array}$$ (5)

$$\begin{array}{c} {\text{3C}}_{\text{3}}\text{A CaS}{\text{O}}_{\text{4}}\;+\text{ 8CaS}{\text{O}}_{\text{4}}\;+\text{ 6CaO}+\text{96}{\text{H}}_{\text{2}}\text{O}=\;\text{3}\left(\text{3CaO}\cdot \text{A}{\text{l}}_{\text{2}}{\text{O}}_{\text{3}}\cdot \text{3CaS}{\text{O}}_{\text{4}}\cdot \text{32}{\text{H}}_{\text{2}}O\right) \end{array}$$ (6)

The active components in steel slag (such as Al_2_O_3_ and SiO_2_) react with Ca(OH)_2_ to consume some water and produce C-S-H gel, which limits the formation of ettringite [22]. The observed reduction in Ca(OH)_2_ and the suggested increase in C-S-H can be explained in Equations (7), (8) and (9):


$$\begin{array}{c} \text{2}{\text{C}}_{\text{3}}\text{S }+\;\text{6}{\text{H}}_{\text{2}}\text{O }\to \text{ C}-\text{S}-\text{H }+\text{ 3Ca}(\text{OH}{)}_{\text{2}} \end{array}$$ (7)

$$\begin{array}{c} \text{2}{\text{C}}_{\text{2}}\text{S}+\;\text{6}{\text{H}}_{\text{2}}\text{O }\to \text{ C}-\text{S}-\text{H }+\text{ Ca}(\text{OH}{)}_{\text{2}} \end{array}$$ (8)

$$\begin{array}{c} \text{Ca}(\text{OH}{)}_{\text{2}}\;+\text{ Si}{\text{O}}_{\text{2}}\;+\;{\text{H}}_{\text{2}}\text{O }\to \text{ C}-\text{S}-\text{H} \end{array}$$ (9)

### 4.4. Fourier-Transform Infrared Spectroscopy analysis

**Fig 17:**
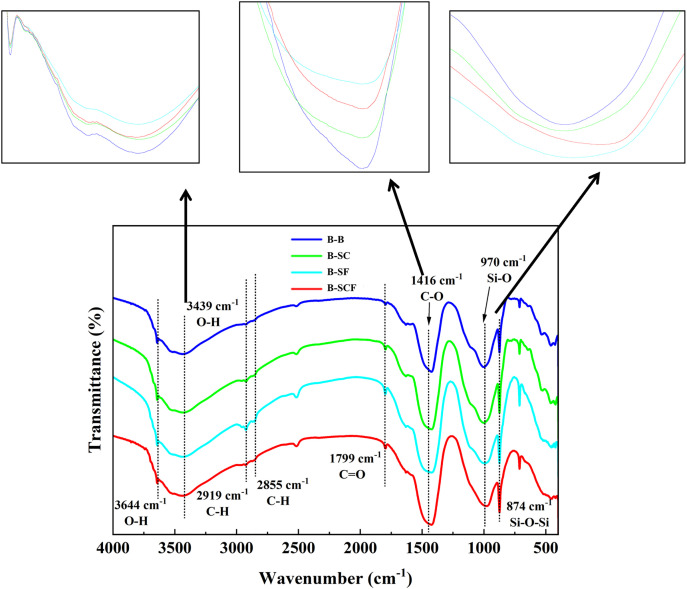
Fourier-Transform Infrared Spectroscopy test results of specimens.

Fig 17 shows that the four groups of specimens had significant absorption peaks at 3,644 cm^-1^, 3,439 cm^-1^, 2,919 cm^-1^, 2,855 cm^-1^, 1,799 cm^-1^, 1,416 cm^-1^, 970 cm^-1^, and 874 cm^-1^. Of these, the absorption peaks at 3,644 cm^-1^ and 3,439 cm^-1^ are typically associated with the O-H stretching vibrations in Ca(OH)_2_, and the absorption peak at 1,416 cm^-1^ is generally related to the C-O stretching vibrations in calcium carbonate. The control-group specimens consistently followed the B-SF < B-SCF < B-SC < B-B sequence, which might indicate that the replacement of traditional sand and gravel with steel slag reduced the concentrations of Ca(OH)_2_ and CaCO_3_ in the concrete. This aligns with the intensity of the Ca(OH)_2_ and CaCO_3_ diffraction peaks in the XRD test results. The absorption bands observed at 2,919 cm^−1^ and 2,855 cm^−1^ are attributed to the asymmetric and symmetric stretching vibrations of C-H bonds, respectively. The absorption peak at 1,799 cm^-1^ typically represents C = O stretching vibrations, which are usually associated with polycarboxylic acid superplasticizers; these results show that the variations between each group had little effect on these substances. The absorption peak at 970 cm^-1^ is generally related to the Si-O stretching vibrations in hydrated calcium silicate; the intensities of absorption peaks exhibited by the various specimens followed the B-B < B-SC < B-SCF < B-SF sequence, which indicates that the increased concentration of hydrated calcium silicate in the concrete was primarily related to the replacement of natural sand with steel-slag fine aggregate. The intensity of the peak was smaller for the B-SCF specimen than for the B-SF specimen; this result may be due to the fact that steel-slag aggregates consumed some water, which weakened the hydration reactions between the steel-slag fine aggregate and the cement paste and resulted in a lower C-S-H concentration. This conclusion corresponds to the greater intensity of the C-S-H diffraction peak of the B-SF specimen in the XRD test results. Moreover, a combination of the macroscopic mechanical, microhardness, SEM, XRD, and FTIR data revealed that, although the C-S-H concentration was greater in the B-SF specimen than in the B-SCF specimen, its macroscopic mechanical strength was slightly lower than that of the B-SCF specimen. The possible reason is that the steel-slag aggregates in the B-SCF specimen may have improved the strength and compactness of the ITZ, which supported the bonding between the aggregates and the cement paste. Thus, the macroscopic mechanical strength of the B-SCF was superior to that of the B-SF.

## 5. Conclusions

This research designed four groups of experiments with different ways of replacing concrete aggregates with steel slag. The differences in mechanical properties of the four groups of specimens were explored through compressive-strength tests, flexural-strength tests, splitting tensile-strength tests and failure morphology analyses. The microstructure was analyzed by means of microhardness, SEM, XRD, and FTIR tests. The conclusions of the research are as follows:

(1) Under the conditions of this comparative test, the macro-mechanical tests yielded the following conclusions: the compressive strength, flexural strength, and splitting tensile strength of concrete prepared by different steel slag substitution methods increased in the order B-B < B-SC < B-SF < B-SCF. Steel slag fine aggregates are superior to steel slag coarse aggregates in enhancing the mechanical properties of concrete. When coarse steel slag is crushed into steel slag fine aggregate to replace concrete fine aggregates, its mechanical strength is greater than that of coarse steel slag replacing concrete coarse aggregates. When steel slag simultaneously replaces both coarse and fine aggregates, the strength of concrete is the highest. However, steel slag possesses much higher water absorption than crushed stone. Even though all concrete mixes were formulated with identical nominal water content, the effective water-cement ratio, workability and compactness cannot be identical across test groups. Accordingly, the above conclusions are confined only to the short-term strength improvement trend obtained under specific experimental conditions, and the limitations of this study will be elaborated in the following Discussion section.
(2) Under the conditions of this comparative experiment, microscopic analysis including microhardness testing, SEM, XRD, and FTIR indicated that steel slag improves the compactness of the ITZ in concrete and supports its bond strength. The observations obtained in this work are generally consistent with the findings reported by Ibrahim, Chunlin and other researchers in the Introduction. According to their conclusions, a plausible explanation is that steel slag accelerates the secondary hydration reaction. More calcium silicate hydrate (C-S-H) gel is formed to fill internal pores, which refines the microstructure and ultimately increases the strength of steel slag concrete. Nevertheless, all microscopic results in this study are dominated by qualitative characterization. Accordingly, the above interpretation is presented merely as a potential mechanism.
(3) The comparative mechanical testing of the four types of steel slag concrete under the conditions of this study revealed that replacing fine aggregate with steel slag aggregate improves mechanical performance more effectively than replacing coarse aggregate with steel slag aggregate. The optimal performance was observed in the B-SCF, where both fine and coarse aggregates were replaced. The underlying reason may be that the incorporation of steel slag fine aggregate is associated with the formation of additional hydration products, specifically C-S-H gel. This not only supports the properties of the ITZ but also increases the intrinsic strength of the cementitious matrix, thereby rendering the cement paste surrounding the coarse aggregate denser and stronger. In contrast, replacing only coarse aggregate with steel slag aggregate primarily strengthens the ITZ, with only a limited effect on the cement paste itself. Consequently, replacing fine aggregate with steel slag fine aggregate is more effective in enhancing the mechanical properties of concrete than replacing coarse aggregate with steel slag coarse aggregate. In the all-steel slag concrete, the coarse aggregate, the surrounding cement paste, and the ITZ are all simultaneously reinforced, which explains why it exhibited the best mechanical performance under the present experimental conditions.

## 6. Discussions

This study investigates the effects of different steel slag aggregate replacement modes on the mechanical properties of concrete. Several limitations encountered during the research are discussed below:

(1) In the conditions of this experiment, all concrete mixtures were prepared with the same water-cement ratio and using aggregates in a dry state. Featuring a rough and porous structure, steel slag exhibits higher water absorption than conventional crushed stone, leading to partial mixing water being absorbed and not participating in hydration. This causes variations in the effective water–cement ratio between groups. Furthermore, even when all aggregates were pre-treated to the saturated surface-dry condition before batching, it cannot be confirmed whether the absorbed water would take part in the subsequent hydration. Meanwhile, the amount of water released by steel slag and crushed stone over time is also unable to remain identical. Consequently, the variations in the actual water-cement ratio across groups exerted an influence on the experimental results of this study.
(2) In this comparative experiment, four groups of specimens were designed using an equal-volume replacement method under the same mixture proportion. However, the water absorption capacity of steel slag is markedly higher than that of ordinary crushed stone. Free water that does not participate in hydration evaporates as the concrete hardens, leaving pores and thereby reducing compactness. On the other hand, the reactive components in steel slag can undergo secondary hydration reactions, generating C-S-H gel that fills pores and thus supports compactness. Therefore, considering the distinct material properties between steel slag and conventional crushed stone, the influence caused by inconsistent compactness across test groups cannot be fully excluded under the present experimental conditions.
(3) All concretes in this comparative experiment were prepared under identical conditions with aggregates in a dry state. Owing to the significantly higher water absorption of steel slag compared with ordinary crushed stone, the amount of free water that remained unreacted inevitably varied among the control groups. This leads to differences in the lubricating effect of free water during concrete mixing and transport, resulting in variations in workability, which further affected disparities in concrete strength. Correspondingly, distinct slump values were measured for each group in this study. This phenomenon indicates that differences in workability exerted a noticeable impact on the experimental results.
(4) The steel slag used in this comparative experiment was subjected to aging pretreatment, and its f-CaO and f-MgO contents as well as water immersion expansion rate met the requirements of the Chinese national standard GB/T 20491–2017. However, according to the review by Yiren and Li (2023), laboratory testing conditions cannot fully simulate the long-term behavior of steel slag in dense concrete matrices, and unstable components in steel slag are heterogeneously distributed within coarse aggregates [23]. Although the curing age of concrete specimens in this study reached 180 days, based on the long-term observation results from Wang et al. (2016) and Wu et al. (2021), the hydration reaction rates of f-CaO and f-MgO are relatively slow, typically requiring 1–2 years to be substantially completed [24–25]. Therefore, the experimental results only reflect the short-term performance of specimens. With the continuous development of long-term stability improvement technology for steel slag, the practical application of steel slag concrete will be increasingly extensive.

In summary, this comparative experiment was designed with four groups of specimens. Within the scope of the specific materials, preparation methods, and specimen molding time used in this study, the following short-term experimental conclusions are drawn: for concrete with different steel slag replacement strategies, the compressive strength, flexural strength, and splitting tensile strength increase in the order B-B < B-SC < B-SF < B-SCF. Despite limitations of this study, the above conclusions, if further validated by future studies, will provide valuable references for further research on steel slag concrete.

## Supporting information

S1 Text X-CT analysis of ordinary crushed-stone concrete specimens and all-steel slag concrete specimens. (DOCX)

S2 Table Macro-mechanical tests data. (XLSX)
