# Supplementary material for: Analysis of mechanical properties and micro-mechanisms of concrete based on the method of using steel slag as aggregate replacement: Macro-mechanical tests and microscopic tests of four typical control groups
Source: PLoS One. 2026 Jul 23;21(7):e0352138. doi: 10.1371/journal.pone.0352138 (PMC13395461; doi:10.1371/journal.pone.0352138)
Supplement: S1 Text — (DOCX) [file pone.0352138.s001.docx]

After conducting X-CT tests on ordinary crushed-stone concrete specimens and all-steel slag concrete specimens, the cement paste part was set as white, the aggregate part as black, and the pore and crack parts as blue. The results of the three-dimensional digital models are shown in Figures A and B.


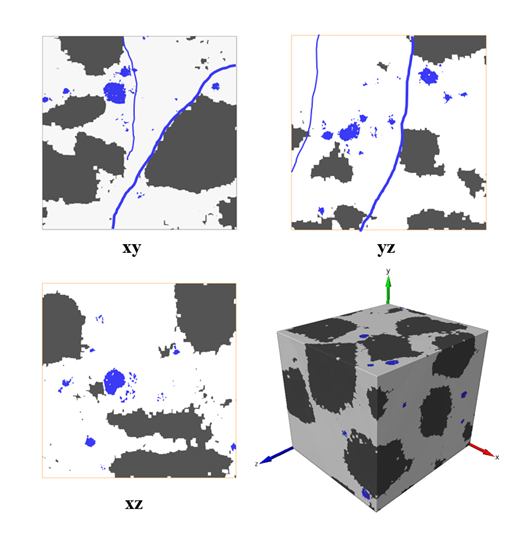


**Fig A. Three-dimensional digital model of ordinary crushed-stone concrete.**


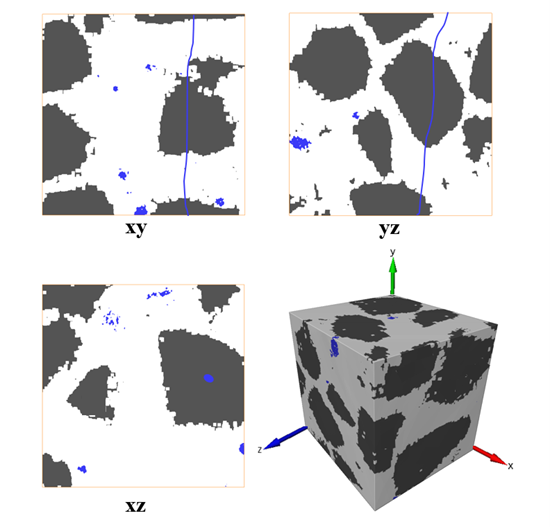


**Fig B. Three-dimensional digital model of all-steel slag concrete.**

As shown in Figures A and B, after being compressed, the ordinary crushed-stone concrete specimens have more cracks and pores, with the cracks mostly occurring at the bonding areas between the cement paste and the crushed stone aggregates as well as within the cement paste, and the pores mostly appearing in the cement paste. In contrast, the all-steel slag concrete specimens have fewer cracks and pores after compression, with the cracks mainly located at the steel slag aggregate distribution areas and fewer pores in the cement paste. This indicates that the steel slag aggregates start to fracture after the concrete specimens are compressed. The difference in the development of cracks and pores between the ordinary crushed-stone concrete specimens and all-steel slag concrete specimens after compression suggests that the adhesion performance between steel slag and cement paste is good, with strong adhesion force. Moreover, steel slag sand can enhance the strength of cement paste. Meanwhile, the X-CT test results correspond to the failure mode of the control group in Section 3.4, supporting the destruction morphology results.
